# Reply to the letter: “A correct diagnosis and close, high-quality follow-up care are crucial for successful management of myasthenia gravis patients”

**DOI:** 10.1055/s-0046-1825752

**Published:** 2026-11-03

**Authors:** Renata Andrade, Wilson Marques Junior, Luiza Vasconcelos, Paula Pungartnik, Thaís Zamboni Berra, Lucas Vieira Cortez, Daiane Beneduzzi

**Affiliations:** 1 Real Hospital Português, Divisão de Doenças Raras, Recife PE, Brazil.; 2 Hospital Pelopidas Silveira, Recife PE, Brazil.; 3 Faculdade de Medicina de Ribeirão Preto, Hospital das Clínicas, Ribeirão Preto SP, Brazil.; 4 Astrazeneca, São Paulo SP, Brazil.; 5 IQVIA, São Paulo SP, Brazil.

Dear Editor,

We appreciate the opportunity to address the questions and valuable feedback provided by the authors of *“A correct diagnosis and close, high-quality follow-up care are crucial for successful management of myasthenia gravis patients”* regarding our article *“Patient journey and treatment pattern in myasthenia gravis: real-world data from the Brazilian public health system”* . 1

The study was conducted using real-world data extracted from administrative records of the Department of Informatics of the Unified Health System (DATASUS, from the Portuguese Departamento de Informática do Sistema Único de Saúde) 2 3 over a 14-year period, comprising more than 13,000 patients diagnosed with myasthenia gravis (MG). As described in the manuscript, our objective was not to evaluate individual clinical management or quality of care, but to provide a population-based overview of treatment trajectories and healthcare utilization within the public health system (SUS, from the Portuguese Sistema Único de Saúde).

We fully acknowledge the clinical relevance of distinguishing serological subtypes of MG, given their differing pathophysiology, prognosis, and therapeutic strategies. However, this level of granularity is not available in DATASUS, which does not store laboratory results, electrophysiological tests, antibody profiles, or functional assessments. 4 Consequently, identifying AChR–positive, MuSK–positive, LRP4–positive, or seronegative MG is not feasible, and neither is differentiating MG from other myasthenic syndromes (e.g., Lambert–Eaton) that may share overlapping diagnostic codes. For this reason, case identification relied exclusively on MG–specific International Classification of Diseases, 10th Revision (ICD–10) codes, an approach consistent with the constraints of administrative data. These limitations, including the impossibility of assessing diagnostic confirmation or serostatus over time, are explicitly acknowledged in the manuscript.

Similarly, the databases do not include clinical information like disease severity, neurological examination findings, remission status, or symptom distribution. Thus, it is not possible to distinguish ocular from generalized MG, evaluate progression patterns, or document clinical outcomes such as minimal manifestation status or pharmacologic remission. The absence of laboratories, electrophysiological, and functional data also precludes assessment of treatment refractoriness, adherence, dosing optimization, or triggers of exacerbation.

Regarding the observed increase in crisis-related hospitalizations over the years, this trend should be interpreted as a reflection of healthcare utilization rather than true changes in disease activity. Improvements in case identification, coding practices, access to specialized care, and the impact of the coronavirus disease 2019 (COVID–19) pandemic likely contributed to the rise in recorded admissions. Furthermore, therapeutic options available within SUS remained limited during the study period, with azathioprine and pyridostigmine being the most frequently identified medications. The databases do not capture oral corticosteroids or newer biologics, which restricts the interpretation of treatment effectiveness and disease stability. Additionally, distinguishing myasthenic from cholinergic crises is not possible in administrative datasets, which lack the clinical descriptors necessary for such subclassification.

With respect to thymectomy, although thymic pathology is clinically relevant and thymoma is known to occur in 10 to 15% of MG cases, 5 DATASUS does not reliably capture surgical indications or link procedures to underlying diagnoses. 4 The procedural codes available do not allow differentiation between thymectomy performed for MG management and other mediastinal surgeries. Including these variables without reliable specificity would introduce substantial misclassification; therefore, thymectomy and thymoma status were not incorporated into the analysis.

Overall, the ambulatory and hospital administrative systems (SIA/SIH, Sistema de Informações Ambulatórias/Sistema de Informações Hospitalares, in Portuguese) used were designed for service billing and operational monitoring. 2 3 They lack essential clinical parameters such as outpatient follow-up intervals, laboratory monitoring, electrophysiological results, documentation of relapses, treatment modifications, or immunosuppressant–related adverse events. 4 Consequently, analyses requiring these elements fall outside the scope of what administrative data can provide.

Despite these inherent limitations, the study offers the first nationwide characterization of the care trajectory of more than 13,000 individuals with MG within SUS. It provides a comprehensive overview of healthcare utilization patterns, hospitalizations, treatment pathways, and temporal trends in disease burden—aspects not previously described on a national level in Brazil and which offer valuable insights into real-world care gaps and system–level challenges. This population–based perspective complements existing clinical evidence and reinforces the need for future studies that integrate administrative data with clinical records to enable more granular assessments of follow-up quality, treatment monitoring, and safety outcomes.

Given that these findings highlight important considerations regarding management patterns, access to care, and continuity of care, a prospective, controlled cohort study is warranted in Brazil. Such an approach would allow validation of hypotheses generated from administrative data and capture clinical nuances that cannot be observed through secondary datasets alone.
